# Prenylated Dihydroflavonol from *Sophora flavescens* Regulate the Polarization and Phagocytosis of Macrophages In Vitro

**DOI:** 10.3390/molecules29194741

**Published:** 2024-10-07

**Authors:** Lu Su, Kairui Rao, Lizhong Wang, Li Pu, Zhijun Zhang, Hongmei Li, Rongtao Li, Dan Liu

**Affiliations:** Center for Pharmaceutical Sciences and Engineering, Faculty of Life Science and Technology, Kunming University of Science and Technology, Kunming 650500, China

**Keywords:** macrophage, polarization, *Sophora flavescens*, inflammation, isopentenyl flavonoids

## Abstract

As an important member of innate immunity, macrophages show remarkable plasticity and heterogeneity, and play an important role in immune regulation, tissue development, homeostasis of the internal environment and injury repair. However, the excessive activation of macrophages is closely related to the occurrence and development of many diseases. The prenylated flavonoid structure is one of the characteristic structures isolated from *Sophora flavescens*, with anti-inflammatory, anti-tumor, anti-allergy and other effects. In this study, the effects of (2*R*)-3*β*,7,4′-trihydroxy-5-methoxy-8-prenylflavanone (TMP), a prenylated dihydroflavonol, on the polarization and phagocytosis of macrophages were systematically studied. In LPS-induced M1-type macrophages, TMP dose-dependently inhibited the expression of COX-2, iNOS and the secretion of NO, IL-1β, IL-6 and IL-18, showing an inhibitory effect on M1 polarization. Further experiments revealed that it was related to the inhibition of TLR4-related AKT/mTOR, MAPK and NF-κB signaling pathways; in IL-4-induced M2-type macrophages, TMP down-regulated the expression of M2-related Arg1, IL-10, TGF-β, CD206 and CD163, as well as the phosphorylation levels of AKT1 and STAT6. For macrophages in a physiological state, it was very important for cells to return from a stress state to a phenotypic stability in the M0 state. These results indicated that TMP negatively regulated the M1/M2 polarization of macrophages, and made them tend to M0 homeostasis, which might provide new theoretical and data support for explaining the anti-inflammatory immunoregulatory activity of *Sophora flavescens*.

## 1. Introduction

Macrophages are an important defense line of the body against foreign invading pathogens, and an important cellular component of the innate immune system with their plasticity and heterogeneity [1]. In addition, macrophages also play an integral role in tissue development, homeostasis and injury repair [2]. Under the influence of different microenvironments in vivo and in vitro, macrophages can differentiate into different phenotypes, enter different polarization states and show differences in function [3]. Different PAMPs (pathogen-related molecular patterns) and DAMPs (damage-related molecular patterns) stimulate macrophages to polarize in different directions, forming classically activated (M1 type) and vicariously activated (M2 type) macrophages [2,4]. M1-type macrophages can induce lipopolysaccharide (LPS) or Interferon-γ (IFN-γ), activate Toll-like receptors (Toll-like receptor, TLR) and IFN pathways, trigger inflammatory responses and release cytokines such as TNF-α, IL-1β, IL-6, IL-23 and IL-27 and some inflammation-related proteins such as cyclooxygenase 2 (COX-2) [5]. M2-type macrophages are mainly induced in Th2 cell immune responses, and can release a large number of anti-inflammatory cytokines such as IL-10 and transforming growth factor β (TGF-β), up-regulate the expression of CD206 and CD163 and promote the elimination of inflammation and repair of tissue damage. These characteristics have been used as markers to distinguish macrophage activity in many studies [3,6,7]. The process of cell polarization can be regulated by a variety of activated pathways. Activation of the JAK2 and mTOR pathway occurs during M1-type polarization, but not during M2-type polarization; further, NF-κB is activated and initiates a series of inflammatory and immune-related responses. MEK/ERK signaling activity is a key regulator of M2-type macrophage polarization, and it also affects inflammation, stress, cell growth, differentiation and death [8,9]. The AKT/mTOR signaling pathway is the main downstream effector of PI3K, [10] and these pathways form a complex network that together regulate the polarization of macrophages. The M1/M2 phenotype is the two extremes of macrophage polarization, which jointly regulates the homeostasis of their internal environment; however, the seemingly opposite functions of M1 and M2 must be strictly regulated in order to make an effective and appropriate response to external stimuli or tissue damage. The overactivation of M1 or M2 macrophages leads to the pathological reaction of many diseases, which also makes them potential therapeutic targets [11].

The dried roots of *Sophora flavescens* Ait. (Leguminosae) are used as a traditional Chinese herb, and are mainly used to treat fever, dysentery, eczema, inflammatory disorders, ulcers and diseases associated with skin burns. Flavonoids and alkaloids are considered to be the main compounds that play biological functions in *Sophora flavescens* [12,13,14]. The role of *Sophora flavescens* in inflammation, immune regulation and tissue repair may be related to abundant flavonoid compounds. Many flavonoids in *Sophora flavescens* have isopentenyl substituents, which often makes it easier for them to penetrate cell membranes and have higher biological activity [14,15]. Our previous screening results on the inhibitory activity of compounds isolated from *Sophora flavescens* showed that flavonoids, especially isopentenyl flavonoids, had a significant inhibitory effect on NO production in macrophages induced by lipopolysaccharide, showing a trend of inhibiting M1 polarization, but at the same time, they often had certain cytotoxicity [16,17]. However, the TMP, a dihydroflavonol with isopentenyl substitutions obtained from *Sophoras flavescens* in the pre-experiment, was found to have significant anti-inflammatory activity and low toxicity. Based on this, we conducted an in-depth study on the effects of TMP on the M1/M2 polarization of macrophages and its regulatory role in macrophage phagocytic function.

## 2. Results

### 2.1. Effects of TMP on M1 Polarization of Macrophages

#### 2.1.1. Effects of TMP on Down-Regulated M1 Polarization of Macrophages

**Figure 1:**
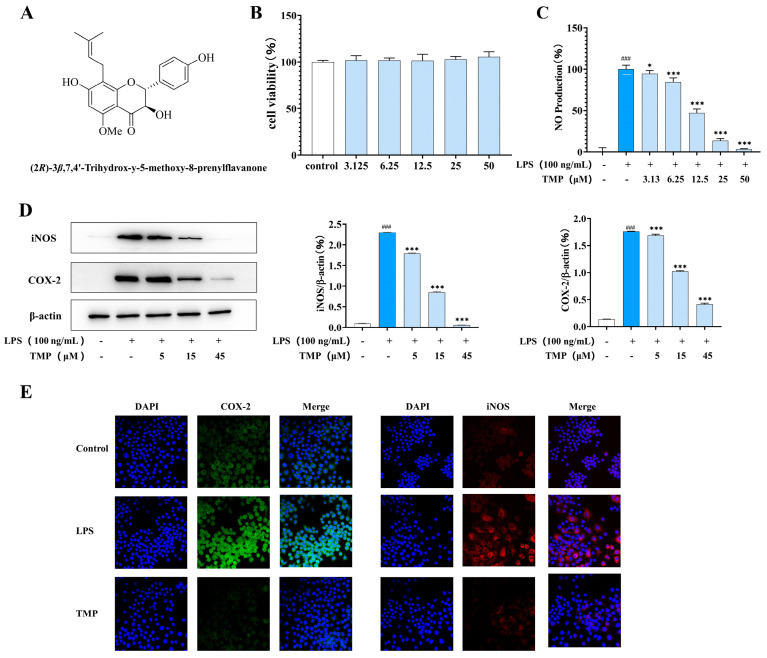
The effects of TMP on M1-type macrophages. (**A**) The structural formula of (2*R*)-3 *β*,7,4′-trihydroxy-5-methoxy-8-prenylflavanone (TMP). (**B**) Cell activity was detected by MTT assay. (**C**) The effect of TMP on NO production was detected by Griess method. (**D**) The effects of TMP on COX-2 and iNOS were detected by western blotting. Actin was probed as an internal control. Compared with the control group, ### *p* < 0.001; compared with the LPS group, * *p* < 0.05, *** *p* < 0.001. (**E**) The effects of TMP on COX-2 and iNOS were detected by fluorescence immunoassay.

The MTT assay was used to detect the effects of compounds on the viability of RAW264.7 cells at different doses (Figure 1B), and the effects of compounds on the production of NO in RAW264.7 cells induced by LPS were detected by Griess method (Figure 1C). The structural formula of TMP is shown in Figure 1A. The MTT assay result showed that TMP had no toxic effect on Raw 264.7 cells in the range of 3.13~50 μmol/L. After LPS induction, macrophages undergo classical activation and polarize into M1-type macrophages. The expression of iNOS and COX-2, two important enzymes associated with inflammation, was significantly increased. As shown in Figure 1D, TMP inhibited COX-2 and iNOS expression in a dose-dependent manner. The results of immunofluorescence (Figure 1E) also showed that TMP treatment reduced the expression of COX-2 and iNOS induced by LPS, which was consistent with the western blot results.

#### 2.1.2. Effects of TMP on M1-Related Cytokines and NLRP3-Related Proteins

**Figure 2:**
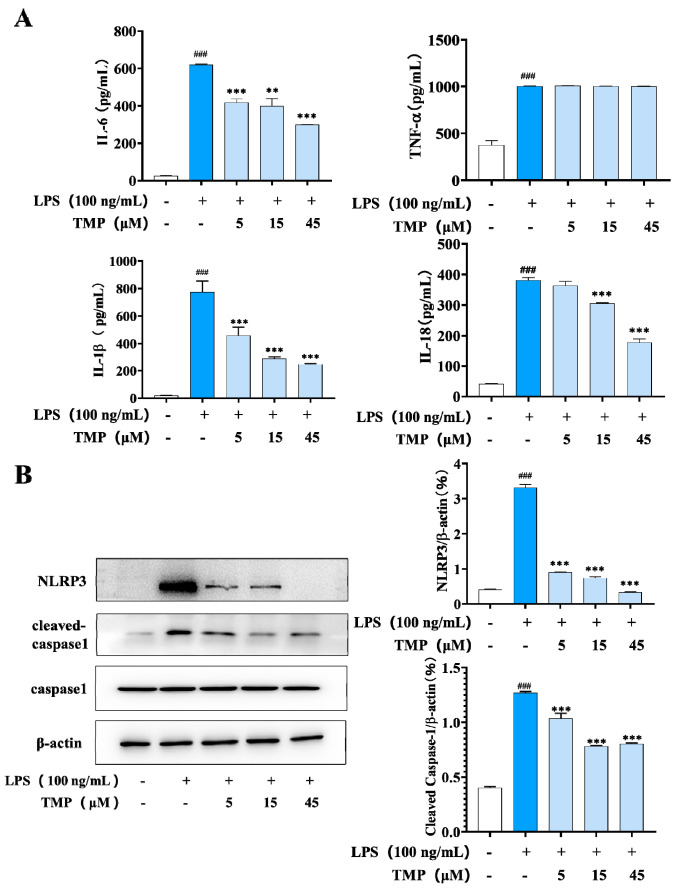
Effects of TMP on M1-related cytokines and NLRP3-related proteins. (**A**) Effects of TMP on IL-6, IL-1β, IL-18 and TNF-α in M1 macrophages; (**B**) Effects of TMP on NLRP3 and its related proteins. Actin was probed as an internal control. Compared with the control group, ### *p* < 0.001; compared with the LPS group, ** *p* < 0.01,*** *p* < 0.001.

M1 macrophages release great amounts of pro-inflammatory cytokines, which drive the development of inflammation. In the process, the multimolecular complex NLRP3 controls the activation of the proteolytic enzyme caspase-1, which regulates the maturation and secretion of cytokines. To further clarify the inhibitory effect of TMP on M1-type macrophages, representative cytokines and NLRP3 and its related proteins were examined. As shown in Figure 2A, LPS up-regulated the secretion of cytokines IL-6, IL-1β, IL-18 and TNF-α, and TMP significantly inhibited the secretion of IL-6, IL-1β and IL-18, but had no effect on TNF-α. Meanwhile, TMP also inhibited the activation of NLRP3 and cleaved caspase-1 in a dose-dependent manner (Figure 2B).

#### 2.1.3. Modulating the M1 Polarization-Related Signaling Pathways in Macrophages

**Figure 3:**
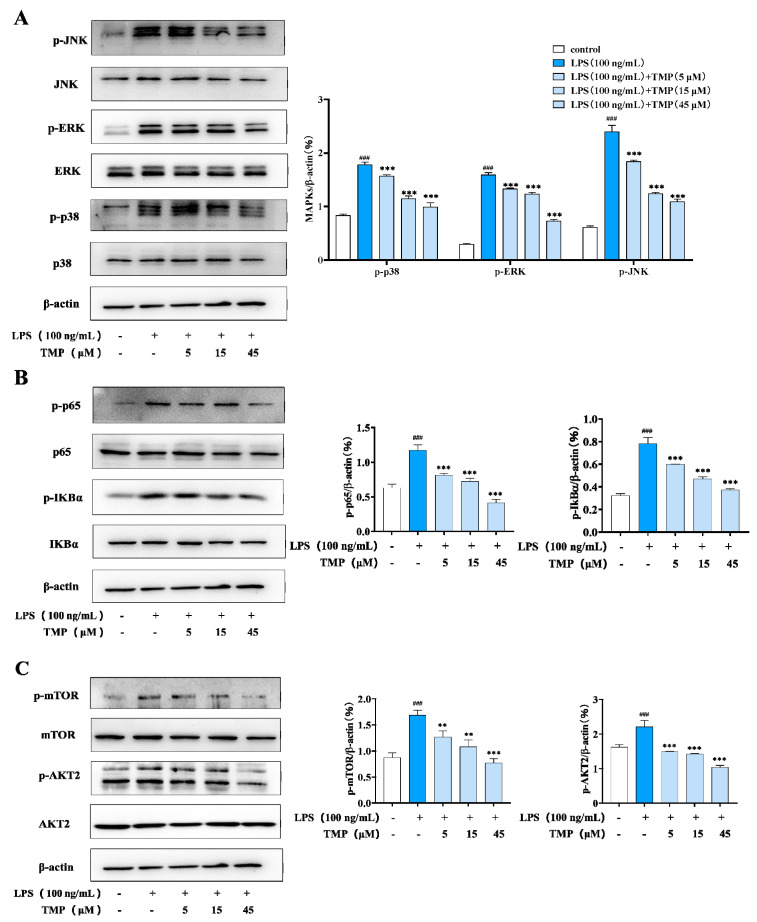
Compounds inhibit the activation of NF-κB, MAPK and Akt/mTOR signaling pathways in M1-type macrophages. (**A**) Effect of TMP on MAKP’s signaling activation; (**B**) Effect of TMP on NF-κB signaling pathway; (**C**) Effect of TMP on the expressions of p-mTOR and p-AKT2 in LPS-stimulated RAW264.7. Actin was probed as an internal control. Compared with the control group, ### *p* < 0.001; compared with the LPS group, ** *p* < 0.01, *** *p* < 0.001.

Multiple pathways are involved in the process of cell polarization. For example, AKT can regulate the differentiation direction of M1/M2 macrophages and regulate the expression of cytokines. To further clarify the regulatory effect of TMP on M1-type macrophages, western blot was used to detect its effect on MAPKs (mitogen-activated protein kinases, MAPKs), NF-κB (nuclear factor kappa B, NF-κB) and AKT/mTOR. As shown in Figure 3A, compared with the control group, LPS treatment activated the MAPK signaling pathway in RAW264.7 cells, and phosphorylation of JNK, P38 and ERK proteins increased significantly. And compared with the LPS group, the above protein phosphorylation levels in the compound-treated group were significantly decreased. NF-κB signaling was also inhibited after treatment with the compound, according to the phosphorylation level of P65 and IκBα. (Figure 3B). Consistent with this, the phosphorylation of mTOR and AKT2 were also reduced (Figure 3C).

### 2.2. TMP Showed an Inhibitory Effect on M2-Type Macrophages

#### 2.2.1. TMP Regulated the Levels of M2 Polarization-Related Proteins and Cytokine

**Figure 4:**
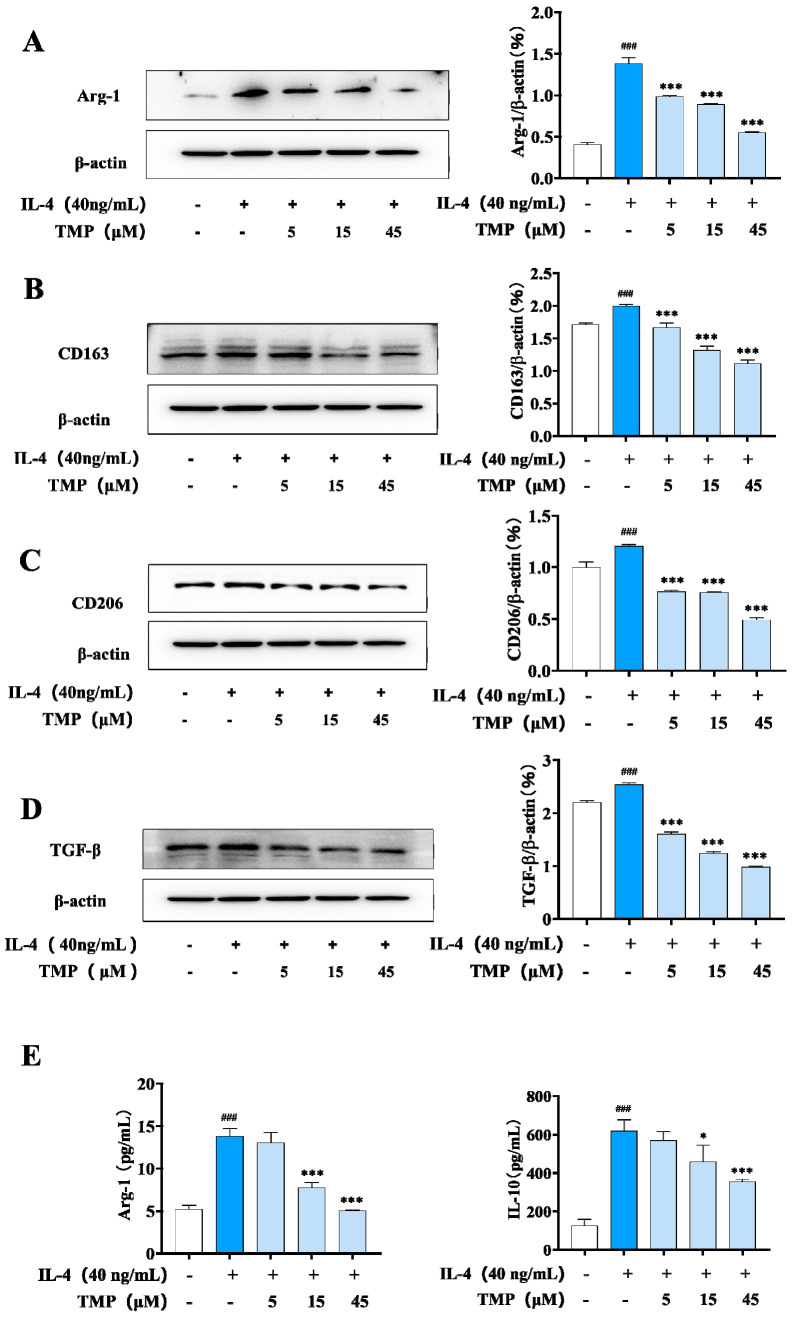
TMP inhibited the expression of related proteins in M2-type macrophages. (**A**) Effect of TMP on Arg-1 expression in M2 macrophages; (**B**) Effect of TMP on CD163 expression in M2 macrophages; (**C**) Effect of TMP on CD206 expression in M2 macrophages; (**D**) Effect of TMP on TGF-β expression in M2 macrophages; (**E**) Effect of TMP on Arg-1 and IL-10 expression; Actin was probed as an internal control. Compared with the control group, ### *p* < 0.001; compared with the IL-4 group, * *p* < 0.05, *** *p* < 0.001.

M2-type macrophages, also known as alternately activated macrophages, can be activated by IL-4/IL-13. In our experiment, macrophages were treated with IL-4 to establish an M2 polarization model of macrophages, in which Arg-1 and IL-10, two markers of macrophage M2 polarization, were highly expressed. IL-4 stimulation also increased the expression of CD163, CD206 and TGF-β (transforming growth factor-β, TGF-β). Compared with the IL-4 group, TMP treatment inhibited the secretion of Arg-1 and IL-10 and the expression of Arg-1, CD163, CD206 and TGF-β (Figure 4), which indicated a significantly inhibitory effect on M2-type macrophages.

#### 2.2.2. TMP Modulated the M2 Polarization-Related Signaling Activation

**Figure 5:**
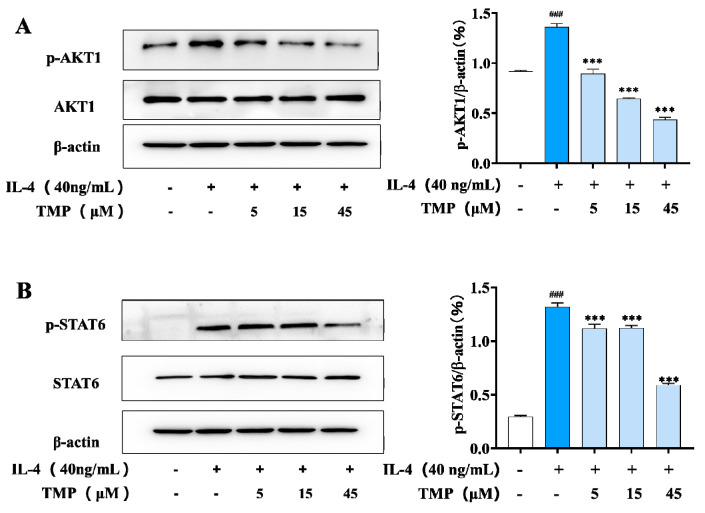
TMP inhibited the expression of related proteins in M2-type macrophages. (**A**) Expression level of AKT1 and its phosphorylation in IL-4-stimulated RAW264.7 cells; (**B**) Expression level of STAT6 and its phosphorylation in IL-4-stimulated RAW264.7 cells. Actin was probed as an internal control. Compared with the control group, ### *p* < 0.001; compared with the IL-4 group, *** *p* < 0.001.

IL-4 activates M2-type macrophages through the JAK1/STAT6 signaling pathway [18], and several genes associated with the M2 macrophage phenotype (Arg-1, CD206 and YM1) are regulated by STAT-6 activity. Studies have shown that activation of the PI3K/AKT1 pathway can promote M2 polarization, thereby enhancing TGF-β expression [6]. Our results showed that IL-4 stimulation significantly increased the phosphorylation levels of AKT-1 and STAT6, whereas TMP treatment significantly inhibited the phosphorylation levels of AKT-1 and STAT6, which showed a negative regulation of M2 macrophages (Figure 5).

### 2.3. The Effect of TMP on M0 Macrophages

**Figure 6:**
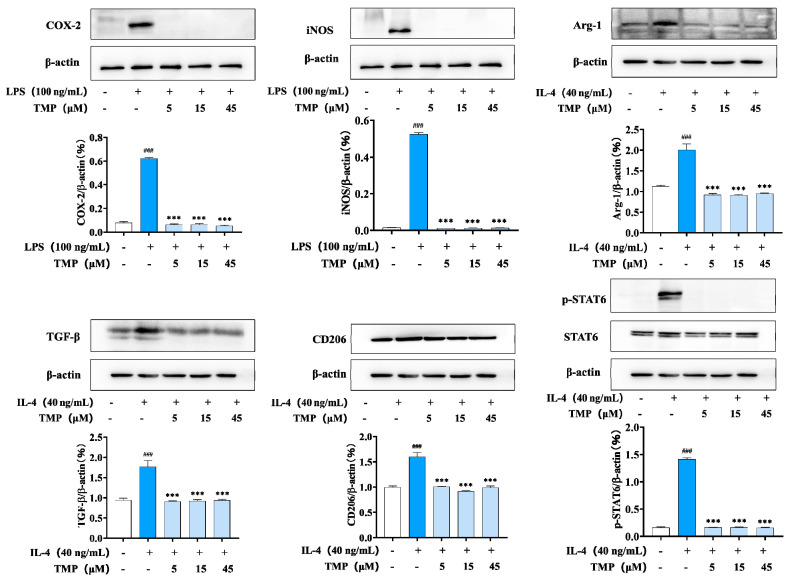
Effects of TMP on M0-type macrophages. The expression levels of iNOS, COX2, TGF-β, CD163, CD206 and ARG-1 were detected using western blotting. The regulation of ERK, STAT6, AKT, PKC and mTOR were detected by western blotting. RAW264.7 cells were exposed to 5 μmol/L AAI for 0–60 min. The expression and phosphorylation of PKC/AKT/ERK were detected by western blotting. Compared with the control group, ### *p* < 0.001; compared with the LPS group or the IL-4 group, *** *p* < 0.001.

Considering the effect of TMP on both M1-type and M2-type macrophages, we detected its effect on M0-type macrophages. M0 represents the physiological state of macrophages when they are not activated or polarized. As shown in Figure 6, TMP did not activate COX-2 and iNOS and also had no significant effect on Arg1 and TGF-β expression in M0-type macrophages. Similarly, TMP treatment had no significant effect on M2-type macrophage-associated CD163 and CD206. In addition, the phosphorylation of STAT6 was not activated after treatment with TMP (Figure 6). These data suggest that TMP does not promote the differentiation of macrophages towards the M1 or M2 type.

### 2.4. The Effect of TMP on Macrophage Phagocytosis

**Figure 7:**
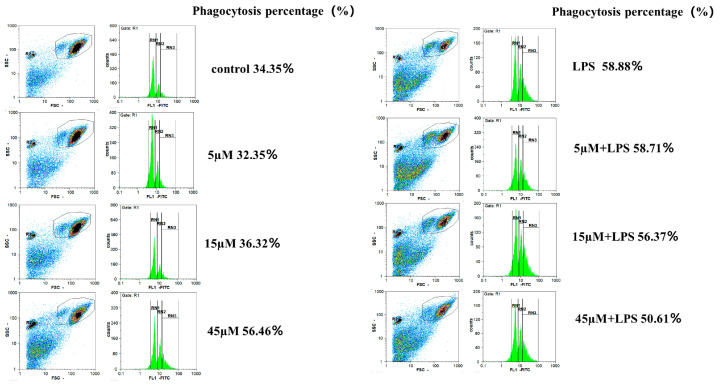
TMP can promote the phagocytosis of macrophages.

Macrophages are sentinel cells of innate immunity, which have phagocytosis functions. Phagocytosis stimulates macrophages to release inflammatory mediators by engulfing and breaking down foreign molecules, thereby triggering the recruitment and activation of other immune cells. Compared with the control group, LPS stimulation promoted the phagocytic function of macrophages. TMP alone can promote the phagocytic capacity of macrophages in a dose-dependent manner. However, the combined effect of TMP and LPS on macrophages did not show this trend (Figure 7).

## 3. Discussion

The polarization of macrophages into different phenotypes is closely related to the microenvironment of macrophages. The dynamic balance between M1-type and M2-type polarization states of macrophages is crucial for maintaining the homeostasis of organism. Once the balance is disrupted, a variety of diseases will occur, including inflammatory bowel diseases, rheumatoid arthritis, cancer [19], atherosclerosis [20], sepsis [21], etc.

The TLR/NFκB, MAPK, PI3K-AKT-mTOR, JAK-STAT and IL-4/STAT6 signaling pathways are commonly involved in the regulation of macrophage polarization [22]. Lipopolysaccharide (LPS) is the main component of the outer membrane of Gram-negative bacteria, and is an inducer of M1 macrophages. After LPS specifically binds to Toll-like receptor 4 (TLR4), it can stimulate IKK kinase through downstream MyD88, and then phosphorylate IκBα to release the binding between IκBα and NF-κB/p65, so that NF-κB/p65 can translocate into the nucleus, play its role as a transcription factor and promote the release of inflammatory factors (IL-1β, IL6, IL-18, TNF-α, etc.) [23,24]. Similarly, the MAPK signaling pathway can also be activated after the binding of LPS and TLR4, which can induce the release of NO by mediating the expression of iNOS and COX-2, and can also mediate the activation of the downstream NF-κB signaling pathway and participate in the secretion of various inflammatory cytokines. This polarizes the cells towards M1 macrophages [25,26]. The LPS-induced RAW264.7 macrophage is a classic model to study the anti-inflammatory mechanism and the signaling pathways related to macrophage activation in vitro [27]. In addition, TLR4 binding to LPS activates PI3K and induces AKT phosphorylation. On the one hand, AKT phosphorylates mTOR to induce the release of IL-6 and TNFα. On the other hand, AKT phosphorylation activates IκB kinase to promote nuclear translocation of NF-κB/p65 to promote the secretion of inflammatory factors and inflammatory mediators [28]. In this study, we found that TMP inhibited the release of NO, IL-1, IL-6 and IL-18, which made it exhibit powerful anti-inflammatory activity, and TMP also inhibited the expression of COX-2, iNOS and NLRP3; it also dose-dependently inhibited the secretion of the inflammatory cytokine IL-6. It was preliminarily demonstrated that TMP inhibited macrophages towards M1-type polarization. The results of the M1-related signaling pathways showed that TMP significantly reduced the phosphorylation levels of JNK, ERK and P38 in LPS-induced RAW26.7 macrophages. At the same time, the phosphorylation of IκBα, p65, AKT2 and mTOR was also inhibited. Similarly, this was accompanied by a reduction in NLRP3 and cleaved caspase-1 activation. NLRP3 promotes inflammation because it activates caspase-1 and promotes the maturation and release of proinflammatory cytokines IL-1β and IL-18. The combination of LPS with TLR4 can activate NF-κB, and the phosphorylation of NF-κB can trigger the activation of NLRP3 and inhibit the expression of TLR4 to achieve the purpose of NLRP3 down-regulation [19]. These data demonstrated the inhibitory effect of TMP on M1-type macrophages. We speculated that TMP might inhibit the secretion of COX-2, iNOS, NO, IL-1β, IL-18 and IL-6 by inhibiting AKT/mTOR, MAPK, NF-κB or a common upstream target, and thus inhibit M1-type macrophage polarization.

IL-4 is an inducer that induces macrophage, which can phosphorylate STAT6 by activating JAKs, thus regulating the expression of several M2-related proteins such as Arg1 and CD206 [3]. It has been reported that the PI3K/AKT1 pathway can promote the polarization of M2-type macrophages, and AKT1 and AKT2 can promote the polarization of M2- and M1-type macrophages, respectively [10]. In this study, we detected that TMP significantly reduced the secretion of IL-10 and TGF-β and the expression of related proteins (Arg-1, CD206, CD163 and TGF-β) in M2-type macrophages. In addition, it also inhibited the phosphorylation of AKT1 and STAT6. Interestingly, when TMP acted on the physiological state of macrophages (M0 state), it did not drive the cell to M1 polarization, nor did it show the characteristics of M2 polarization, but rather stabilized the cell in the M0 state, which is beneficial for maintaining the stability of the physiological state of macrophages. It is currently unclear whether TMP regulates different polarization states of macrophages by targeting the same or completely different targets, which will be the focus of our future research.

**Figure 8:**
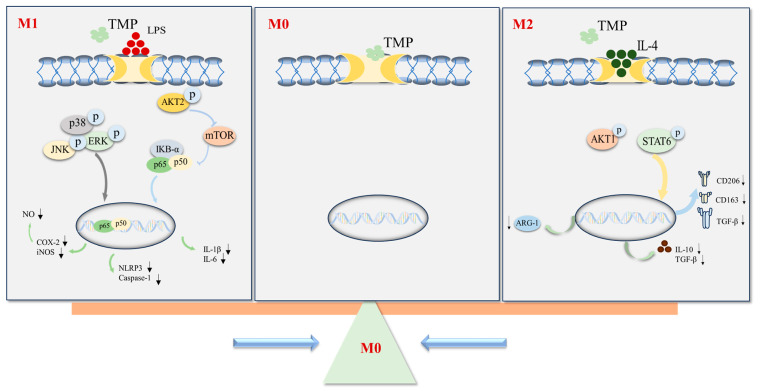
The possible mechanism of TMP on the polarization of macrophages.

In conclusion, the multiple regulatory effects of isopentenyl flavonoids TMP on macrophages were studied in this paper. We suggested that TMP inhibited the polarization of M1-type macrophages by inhibiting the activation of TLR4-related AKT/mTOR, MAPK and NF-κB signaling pathways, and it also inhibited the polarization of M2-type macrophages accompanied by activating AKT1/STAT6 signaling pathways (Figure 8). The above results hinted that macrophages pulled back from the extreme polarization of M1 and M2 macrophages, which helped to regulate the polarization state between M1 and M2. That provides a new therapeutic idea for the treatment of diseases caused by an imbalance in macrophage polarization. TMP increased the phagocytosis of RAW264.7 cells in a dose-dependent manner.

## 4. Materials and Methods

### 4.1. Plant Extraction and Compound Isolation

The dried roots of *S. flavescens* were collected in Honghe Yunnan in June 2019 and identified by Dr. Xuan-Qin Chen. The specimen number is KUMST20190628 and the roots were deposited at the Key Laboratory of Phytochemistry (Kunming University of Science and Technology). TMP was extracted and isolated from them.

The dried roots of *S. flavescens* (20.0 kg) were extracted with 95% EtOH (3 × 40 L, 24 h, each). The extract was distilled under reduced pressure to remove the solvent to obtain an extract. The obtained extract was dissolved in water and then extracted with ethyl acetate three times to finally obtain an ethyl acetate phase (493.0 g). The ethyl acetate phase was separated and enriched by macroporous resin to obtain 308 g of total flavonoids. The total flavonoid extract (300 g) was segmented through a normal phase silica gel column and eluted with a petroleum ether-ethyl acetate (4:1-1:1) gradient to obtain nine polar segments F1~F9 [16].

F6 (92.0 g) was separated by the silica gel column, eluted with a chloroform-methanol (80:1-30:1) gradient and analyzed by TLC, and then semi-prepared by HPLC (50%, MeOH-H_2_O, 3 mL/min) to obtain the compound (2*R*)-3*β*,7,4′-trihydroxy-5-methoxy-8 -prenylflavanone (4.2 mg, 95% purity). The structures were elucidated by NMR techniques, as well as by comparison with the literature. Subsequent articles will use TMP as a substitute name; its CAS number is 204935-85-3.

### 4.2. Cell Culture and Treatment

Mouse macrophage RAW264.7 was purchased from the Kunming Cell Bank of Chinese Academy of Sciences (KCB200603YJ) and incubated in Dulbecco’s Modified Eagle Medium (Gibco, Thermo Fisher, Waltham, MA, USA) supplemented with 10% fetal bovine serum (FBS; Gibco, Thermo Fisher, USA), penicillin (100 U/mL) and streptomycin (100 µg/mL, Solarbio Life Sciences, Beijing, China) at 37 °C in a 5% CO_2_ incubator. LPS (Escherichia coli 055: B5; Sigma-Aldrich, St. Louis, MO, USA) induced polarization of RAW264.7 cells towards the M1 type, while IL-4 (Beyotime, Shanghai, China) induced polarization of the cells towards the M2 type.

### 4.3. Cell Viability Assay

The cell viability was determined using the colorimetric MTT assay. RAW264.7 cells were seeded in 96-well plates (Corning Incorporated, Corning, Somerville, MA, USA) at 3 × 10^4^ cells/well for 24 h and then treated with different concentrations (3.13, 6.25, 12.5, 25 and 50 µmol/L) of the compound for 24 h. Then, cells were incubated with a 20 µL/well of MTT solution (5 mg/mL, Solarbio Life Sciences, Beijing, China) at 37 °C for 3.5 h. DMSO (Solarbio Life Sciences, Beijing, China) was added to dissolve the purple formazan and incubated at room temperature for 10 min, and the absorbance at 490 nm was measured using a microplate reader (Thermo Fisher Scientific, Waltham, MA, USA).

### 4.4. Analysis of NO Production

RAW264.7 cells were seeded in 96-well plates at 4 × 10^4^ cells/well for 24 h and then treated with different concentrations (3.13, 6.25, 12.5, 25 and 50 µmol/L) of the compound in the presence of LPS (100 ng/mL) for 24 h. The supernatants were collected and treated with Griess reagent (Beyotime Institute of Biotechnology, Shanghai, China), and incubated at room temperature for 10 min. The absorbance at 540 nm was measured using a Thermo Fisher Scientific microplate reader (Waltham, MA, USA).

### 4.5. Enzyme-Linked Immunosorbent Assays

RAW264.7 cells were seeded in 12-well plates at 3 × 10^5^ cells/well and then incubated for 24 h. The cells were treated with LPS (100 ng/mL) or IL-4 (40 ng/mL) to construct the M1/M2 cell polarization model. Different concentrations (5, 15 and 45 µmol/L) of the compound were incubated for 24 h. The expression levels of TNF-α, Il-1β, IL-6 and IL-10 in cell culture supernatant liquid and Arg-1 in cell lysate were quantified using mouse ELISA kits (Novus Biologicals, Englewood, CO, USA, Lot: 486956, 834472, 602865, Novus Biologicals, USA, Lot: 772051, CUSABIO, Wuhan, China, Lot: J22012927) according to the manufacturer’s instructions.

### 4.6. Western Blot Analysis

The administration treatment was the same as “2.6”: RAW264.7 cells (M0) were seeded in 12-well plates at 3 × 10^5^ cells/well for 24 h, and then treated with different concentrations (5, 15 and 45 µmol/L) of the compounds for 24 h. The protein samples were obtained from the lysate of cultured cells and the protein concentration was determined. Proteins were separated by 10% sodium dodecyl sulfate-polyacrylamide gel electrophoresis (SDS-PAGE) and then transferred to PVDF (polyvinylidene fluoride) membranes (Millipore, Burlington, MA, USA). After this, they were blocked by 5% skim milk in TBS containing 0.1% Tween 20 (TBS-T) for 2 h, with specific primary antibodies (iNOS, COX-2, NLRP3, caspase-1, cleaved caspase-1, JNK, p-JNK, p38, p-p38, ERK, p-ERK, Cell Signaling Technology, Boston, MA, USA; Arg-1, CD163 Beyotime, Shanghai, China, AF1381, AF6453; CD206, Invitrogen-Thermo Fisher, Waltham, MA, USA) at 4 °C overnight. After washing by TBST and PBS, the PVDF membranes were incubated with an HRP-conjugated secondary antibody (Beyotime, Shanghai, China, A0208), and detected by using the Ultra-sensitive ECL chemiluminescent substrate (Biosharp, Hefei, China).

### 4.7. Fluorescence Staining

Macrophages were fixed with 4% paraformaldehyde for 15 min at room temperature after administration, and then washed with PBS for four times. The primary antibodies (COX-2, iNOS) were added to the cells at 4 °C overnight. After washing with PBS, the cells were incubated with the secondary antibody, and the stained images were captured under a laser scanning confocal microscope (Nikon A1 AIR MP+, Tokyo, Japan).

### 4.8. Phagocytosis Experiment

RAW264.7 cells were seeded in 12-well plates at 3 × 10^5^ cells/well for 24 h. The cells were treated with different concentrations (5, 15 and 45 µmol/L) of the compounds for 2 h and then incubated for 24 h with or without LPS (100 ng/mL). After carefully washing twice with 1 mL of PBS per well, 1.5 mL of DMEM and 100 μL of fluorescent microspheres were added into each well (prepared in advance according to the instructions). After the reaction, the supernatant was discarded. After being carefully cleaned twice with 1 mL PBS in each well, the cells were collected into 1.5 mL EP tubes. After being blown evenly with 1 mL PBS in each tube, the cells were analyzed by flow cytometry.

Phagocytic percentage (%) = phagocytic macrophages/total number of macrophages ×100%.

### 4.9. Statistical Analysis

Statistical analysis was performed by using SPSS 23.0 (SPSS Inc., Chicago, IL, USA) software. *p*-values were assessed using one-way analysis of variance (ANOVA), and the data shown are representative of at least triplicate independent experiments. When the *p* < 0.05, the data were considered to be statistically significant. When *p* < 0.05, it is represented by the symbol * or #; when *p* < 0.01, it is represented by the symbol** or ##; when *p* < 0.001, it is represented by*** or ###.

## Data Availability

The datasets used in the present study are available from the corresponding authors upon reasonable request.

## Abbreviations

LPS	Lipopolysaccharides
COX-2	Cyclooxygenase-2
iNOS	Inducible nitric oxide synthase
IL-1β	Interleukin-1beta
IL-4	Interleukin-4
IL-6	Interleukin-6
IL-10	Interleukin-10
IL-18	Interleukin-18
AKT/mTOR	Protein kinase B/mammalian target of rapamycin
MAPK	Mitogen-activated protein kinase
NF-κB	Nuclear factor kappa-light-chain-enhancer of activated B cells
Arg1	Arginase 1
TGF-β	Transforming growth factor beta
CD163	Scavenger receptor cysteine-rich type 1 protein M130
CD206	Mannose Receptor
STAT6	Signal Transducer and Activator of Transcription 6
MEK/ERK	Methyl Ethyl Ketone/extracellular regulated protein kinases
PI3K	Phosphoinositide 3-kinase
MTT	3- (4,5-Dimethyl-2-Thiazolyl)-2,5-Diphenyl Tetrazolium Bromide
NLRP3	Nucleotide-binding oligomerization domain, leucine-rich repeat and pyrin domain-containing 3
TNF-α	Tumor Necrosis Factor-α
JNK	c-Jun N-terminal kinase
IκBα	Inhibitor of kappa B alpha
IKK	Inhibitor of kappa B kinase
